# The HYLAN M Study: Efficacy of 0.15% High Molecular Weight Hyaluronan Fluid in the Treatment of Severe Dry Eye Disease in a Multicenter Randomized Trial

**DOI:** 10.3390/jcm9113536

**Published:** 2020-11-02

**Authors:** Gysbert-Botho van Setten, Christophe Baudouin, Jutta Horwath-Winter, Daniel Böhringer, Oliver Stachs, Ebru Toker, Sultan Al-Zaaidi, Jose M. Benitez-del-Castillo, Ria Beck, Osama Al-Sheikh, Berthold Seitz, Stefano Barabino, Herbert A. Reitsamer, Wolfgang G.K. Müller-Lierheim

**Affiliations:** 1 Karolinska Institutet, Department of Clinical Neuroscience, St. Eriks Eye Hospital, 11282 Stockholm, Sweden; gysbert.van.setten@ki.se; 2 Quinze-Vingts National Eye Hospital & Vision Institute, IHU Foresight, 75571 Paris, France; cbaudouin@15-20.fr; 3 Department of Ophthalmology, Medical University Graz, 8036 Graz, Austria; jutta.horwath@medunigraz.at; 4 Eye Center, University Eye Hospital Freiburg and Medical Faculty, Albert Ludwigs University, 79106 Freiburg, Germany; daniel.boehringer@uniklinik-freiburg.de; 5 Department of Ophthalmology, University Medical Center Rostock, 18057 Rostock, Germany; oliver.stachs@uni-rostock.de (O.S.); ria.beck@gewebenetzwerk.de (R.B.); 6 Department of Ophthalmology, Marmara University School of Medicine, 34899 Istanbul, Turkey; dretoker@gmail.com; 7 Department of Ophthalmology, PSMMC Prince Sultan Military Medical City, MSD Medical Services Department, MODA Ministry of Defense and Aviation, Riyadh 12233, Saudi Arabia; alzaaidi_s@yahoo.com; 8 Universidad Complutense de Madrid, Hospital Clinico San Carlos, Clinica Rementeria, 28040 Madrid, Spain; jbenitez.hcsc@salud.madrid.org; 9 KKESH–King Khaled Eye Specialist Hospital, Riyadh 11462, Saudi Arabia; oshaikh@kkesh.med.sa; 10 Department of Ophthalmology, Saarland University Medical Center, 66421 Homburg/Saar, Germany; berthold.seitz@uks.eu; 11 Ocular Surface & Dry Eye Center, Ospedale L. Sacco, University of Milan, 20157 Milan, Italy; stebarabi@gmail.com; 12 Department of Ophthalmology & Department of Experimental Ophthalmology and Glaucoma Research, University Clinic Salzburg, Paracelsus Medical University, 5020 Salzburg, Austria; h.reitsamer@salk.at; 13 CORONIS GmbH, 81241 Munich, Germany

**Keywords:** dry eye disease, severe keratitis, hyaluronan, hylan A, multicenter, randomized trial

## Abstract

The aim of the HYLAN M study was to investigate if symptoms and/or signs of patients suffering from severe dry eye disease (DED) can be improved by substituting individually optimized artificial tear therapy by high molecular weight hyaluronan (HMWHA) eye drops. In this international, multicenter study, patients with symptoms of at least ocular surface disease index (OSDI) 33 and corneal fluorescein staining (CFS) of at least Oxford grade 3 were included. A total of 84 per-protocol patients were randomized in two study arms. The control group continued to use their individual optimum artificial tears over the study period of eight weeks; in the verum group, the artificial tears were substituted by eye drops containing 0.15% HMWHA. At the week 8 visit, the average OSDI of the verum group had improved by 13.5 as compared to the control group (*p* = 0.001). The best corrected visual acuity (BCVA) had improved by 0.04 logMAR (*p* = 0.033). CFS, tear film break-up time (TBUT), Schirmer I, lid wiper epitheliopathy (LWE), mucocutaneous junction (Yamaguchi score), and tear osmolarity were not significantly different between the verum and control groups (*p* > 0.050). We conclude that for most patients with severe DED, 0.15% HMWHA eye drops provide excellent improvement of symptoms without impairment of dry eye signs.

## 1. Introduction

Dry eye disease (DED) is a multifactorial disorder affecting 5% to 35% of the world population [1]. In cases of severe DED, patients experience symptoms of ocular discomfort and visual instability, resulting in a considerable loss of quality of life. Ocular burning or stinging, ocular discomfort, and ocular pain are rated as the most important symptoms by patients [2]. The loss of visual stability additionally causes a negative impact on quality of life and is attributed to an instable tear film [3]. DED has a significant socio-economic effect due to considerable direct treatment costs as well as indirect costs due to a loss of work productivity [4,5,6].

The Tear Film and Ocular Surface Society (TFOS) summarizes the current concepts as a staged management and treatment for DED. Lubricating, hydrating eye drops not targeting the underlying pathophysiology of DED are the standard long-term treatment for DED [7]. Secretagogues may have an initial effect, but this may fade over time. If ocular lubricants and secretagogues are not providing acceptable relief from symptoms, immunomodulatory eye drops such as cyclosporine may be tried [7]. This treatment approach reflects the current model of pathophysiology of DED. Other models can either reflect a different understanding or the existence of regional differences. The Asia Dry Eye Society, e.g., proposes a tear film-oriented therapy distinguishing between lipid layer, aqueous/secretory mucin deficiency, and corneal epithelial surface/membrane bound mucin deficiency [8,9]. It is assumed that the instability of the tear film increases the friction between the eyelids and the eye, which will result in ocular inflammation and epithelial damage [9,10,11]. Persistent systemic conditions of the patient or environmental adverse conditions may lead to a self-maintaining vicious circle of inflammation, which may result in chronic, eventually irreversible forms of severe dry eye [12]. Hence, a prerequisite for successful therapy is personalized clinical assessment and treatment [13]. Inflammation caused by autoimmune diseases or elevated osmolarity of the tear film due to excess evaporation have been pointed out as driving forces of the vicious circle of inflammation. Here, the treatment of inflammation as a driving force within the vicious circle plays a key role [14,15,16]. Although hyperosmolarity measured in the tear meniscus has been proposed for diagnosing DED, the level of osmolarity and its absolute value apparently plays a minor role as a local stress factor compared to the extent of diurnal variation [17,18,19]. Severe discomfort as a symptom of DED may transit to changes in neuroception, reflecting nerve damage. Nerve damage itself may be another underlying pathophysiological mechanism in severe chronic DED [20]. Not only does nerve damage result in a reduction of trophic support for the corneal epithelium, it could also initiate and maintain inflammation, and thus the interplay between nerve damage and inflammation needs to be taken into consideration [21,22]. This might contribute to the well-known discordance between dry eye signs and symptoms [23,24,25,26]. Although some substances have recently demonstrated certain potential in the treatment of neuropathic keratopathy, there is currently no therapy that directly addresses the underlying nerve damage [27,28]. Additionally, patients suffering from neuropathic ocular pain frequently respond poorly to treatment with lubricant eye drops [29,30].

High molecular weight hyaluronan (HMWHA) has in contrast to the majority of lubricating eye drops anti-inflammatory activity and the capability to reduce the activity of the pain transducing channel TRPV1 in nociceptive nerves, thus reducing neuropathic pain [31,32,33,34,35]. HMWHA in eye drops provides excellent lubrication due to shear-thinning properties such as the natural tear film, good hydrating and water-binding properties resulting in reduced evaporation, and stabilization of the ocular surface barrier function to recover the protection against infection [36]. A recent study demonstrated in an environmental dry eye stress model in mice that HMWHA eye drops protect the ocular surface from mechanical damage and inflammation better than low molecular weight hyaluronan (LMWHA) [37].

The aim of the presented study (HYLAN M study) was to investigate the effect of HMWHA eye drops in comparison with other tear substitutes in an international multicenter prospective open label clinical investigation.

## 2. Experimental Section

### 2.1. Study Design

The HYLAN M study, a multicenter prospective randomized open label study, was performed in 11 centers in eight countries. Details of the study centers, administrative structure, planning, and conduct are provided in Appendix A. The study adhered to the Declaration of Helsinki, was approved by ethics committees of all eight countries involved, and registered as outlined in Appendix B.

Patients identified as having severe DED were randomized in two parallel arms. The control group continued with the currently used therapy as by the time of inclusion. In the verum group, the individual lubricant eye drops used by each patient by the time of inclusion were replaced by preservative-free eye drops containing 0.15% HMWHA dissolved in isotonic saline solution buffered with 120 mmol/L phosphate (Comfort Shield^®^ eye drops; i.com medical GmbH, Munich, Germany; see Appendix C). Concomitant treatment for dry eye such as cyclosporine eye drops remained unchanged in both arms.

Demographic data and medical history were recorded during the baseline visits. Symptoms and signs associated with DED were assessed at the baseline visit and at the week 4 and week 8 follow-up visits, respectively (see Table 1).

### 2.2. Participants

Patients over 18 years suffering from DED of any underlying etiology were eligible for inclusion. The patients had to be under stable, unchanged dry eye therapy for at least two months (in case of concomitant cyclosporine therapy three month) by the time of inclusion. Patients were excluded if they participated in any other clinical trial, suffered from eye diseases other than dry eyes, had ocular surgery less than three months prior to study inclusion, were using punctual plugs, or had masquerading conditions as identified by Karpecki [38]. Masquerading conditions are conjunctivochalasis, recurrent corneal erosions, epithelial basement membrane dystrophy, mucin fishing syndrome, floppy eyelid syndrome, giant papillary conjunctivitis, Salzmann’s nodular degeneration, and ocular rosacea.

As inclusion criteria for severe dry eye, the primary criteria according to Baudouin et al. were chosen [39]. The dry eye symptoms were assessed using the ocular surface disease index (OSDI) questionnaire [40]. For inclusion, patients had to have an OSDI score of 33 or more [39]. As dry eye sign, corneal fluorescein staining (CFS) was chosen [41]. For inclusion, patients had to have at least one eye with CFS Oxford grade 3 or more, but no confluent CFS.

Based on the OSDI score and visually assessed CFS grade, the study centers decided on preliminary enrollment. CFS images were transferred to the reading center for quantitative electronic evaluation of corneal fluorescein staining (RC1), as described in Appendix D. The reading center was masked for the assigned treatment to minimize bias. If the submitted images met the criteria for automated assessment, the staining was not confluent, and at least one eye of the patient met the CFS inclusion criteria, the reading center confirmed the definite enrollment of the patient. Otherwise the patient was excluded as “screen fail”.

The eyes with the higher staining score were defined as study eyes. However, the fellow eye was retrospectively redefined as a study eye if the masked reading center determined that the images from the fellow eye had significantly better contrast than those of the designated eye, given that the follow eye fulfilled all inclusion criteria at the baseline visit.

### 2.3. Efficacy Assessment

TFOS recommends to formally assess dry eye disease by symptoms using a questionnaire such as the OSDI in combination with at least one test for homeostasis markers (signs) such as tear break-up time (TBUT), tear osmolarity, or ocular surface staining [42]. Accordingly, the OSDI questionnaire was used to assess dry eye symptoms throughout the HYLAN M Study. Due to the variable and controversial association between dry eye signs and symptoms, the following standardized battery of tests for dry eye signs was applied in the HYLAN M study. Best corrected visual acuity (BCVA) was used as an indicator of visual stability. CFS, TBUT, and the Schirmer test without topical anesthesia (Schirmer I) were used to assess the lubricating properties, stability, and quantity of the tear film [43,44,45,46,47]. Tear film osmolarity in the lower tear meniscus was additionally measured using the TearLab osmolarity system (TearLab Corporation, San Diego, CA, USA). Specific training of the correct test performance and daily calibration of the test instrument was performed at every study center. Intraocular pressure (IOP) was determined by Goldman applanation tonometry or Icare^®^ tonometry at the baseline and week 8 visits as a safety parameter to rule out uncontrolled glaucoma or ocular hypertension.

Additionally, most but not all study centers additionally performed lissamine green staining of the lid rim to assess lid wiper epitheliopathy (LWE) as the Korb score and the mucocutaneous junction (Marx line) as the Yamaguchi score [48,49,50]. Moreover, four out of 11 study centers assessed the subbasal nerve plexus by confocal laser scanning microscopy and provided images to a second masked reading center (RC2) for evaluation [51].

Table 1 provides the testing schedule of the HYLAN M study. For more details on diagnostic test methods and assessment of results by reading centers, see Appendix A and Appendix D.

### 2.4. Statistical Analysis

The primary endpoint for the demonstration of superiority of Comfort Shield eye drops (verum group) over other ocular lubricants currently used by the patients (control group) was the difference between CFS at week 8 and at baseline visits as quantitatively determined by RC1 (see Appendix D).

The key secondary endpoint was the difference between OSDI scores at week 8 and baseline [40]. To further analyze the improvement of symptoms, OSDI subscores for the questions related to pain OSDI_pain_ (OSDI questions 1–3) and OSDI subscore for questions related to visual stability OSDI_vision_ (OSDI questions 4–9) were analyzed according to the following formulas:

(1) $${\mathrm{OSDI}}_{\mathrm{pain}}=\frac{\mathrm{sensitive}\;\mathrm{to}\;\mathrm{light}+\mathrm{feeling}\;\mathrm{gritty}+\mathrm{pain}\;\mathrm{score}\;\mathrm{eye}}{n}\times \;25$$

(2) $${\mathrm{OSDI}}_{\mathrm{vision}}=\frac{\mathrm{blurred}\;\mathrm{vision}+\mathrm{poor}\;\mathrm{vision}+\mathrm{reading}+\mathrm{driving}\;\mathrm{at}\;\mathrm{night}+\mathrm{computer}\;\mathrm{ATM}+\mathrm{watch}\;\mathrm{TV}}{n}\times 25$$

*n* = number of questions answered (at most, 3 for the pain, and 6 for vision subscores, respectively)

Additional secondary endpoints were the differences between BCVA, TBUT, Schirmer I value, tear osmolarity, Korb score, and Yamaguchi score at week 8 and baseline, respectively.

The full analysis set (FAS) was defined as all patients who were not in “screen fail” status, have at least once used their eye drops, have data for the primary endpoint (CFS from reading center RC1) or the key secondary endpoint OSDI, and have had at least one follow-up visit. The per-protocol set (PPS) comprised all patients of the FAS without any major protocol deviation that could substantially affect the evaluation of the randomized treatment. One patient who had not taken lubricant eye drops before the time of inclusion and patients who did not show up for the week 8 follow-up visit were excluded from the PPS.

Considering the potential influence of climatic differences, two subgroups of the verum and control groups were defined: Desert = all patients from the two study centers in Riyadh, Saudi Arabia, and Europe = all other patients. For these subgroups, the results of CFS and OSDI were separately analyzed [52].

## 3. Results

### 3.1. Participant Flow

**Figure 1:**
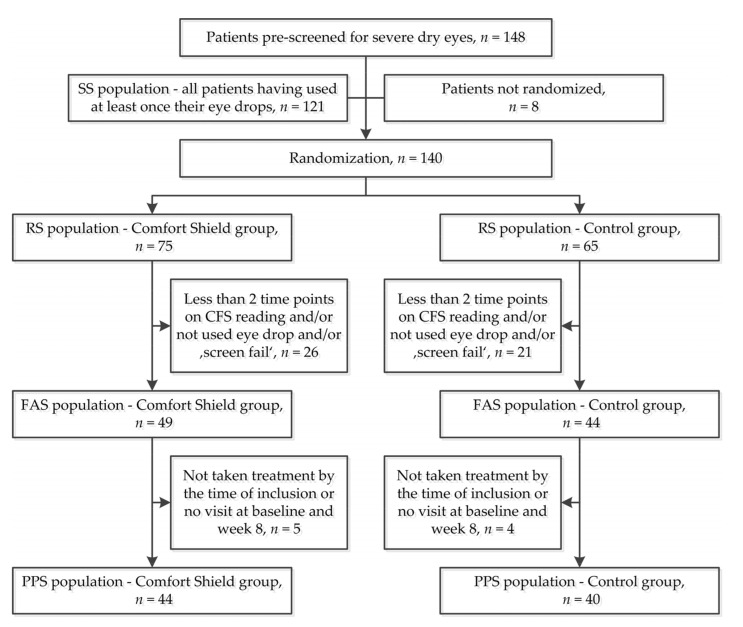
Participant flowchart.

Figure 1 summarizes the participant flow. In total, 148 patients were pre-screened for severe dry eye disease. Out of these, eight patients were excluded at the initial interview and not randomized, and 121 patients have at least once taken their eye drops (= safety set SS). In total, 140 patients were preliminarily included by the study centers and randomized (= randomized set RS), out of these 75 in the Comfort Shield group and 65 in the control group. Out of the 140 randomized patients, 47 were classified as “screen fails” by the reading center or did not use their eye drops. Of the remaining 93 patients, 49 had been randomized to the Comfort Shield group and 44 had been randomized to the control group (=full analysis set FAS). Out of these, five patients of the Comfort Shield group and four patients of the control group did not show up for the week 8 visit. Therefore, the per-protocol set (PPS) comprises 44 patients in the Comfort Shield group and 40 patients in the control group (see Figure 1).

### 3.2. Demographic Data

The full analysis set (FAS) of the HYLAN M study comprises 93 patients. Out of these, 84 belonged to the per-protocol set (PPS), 44 belonged to the Comfort Shield group, and 40 were in the control group. The study was performed in different climate zones, and patients from different ethnicities were enrolled. Table 2 provides an overview of the socio-demographic data of the study.

An overview of the medical history for the PPS set is provided in Appendix E, Table A1.

### 3.3. Efficacy Results

The results presented below refer to the 84 per-protocol patients in the study (PPS population in Figure 1).

#### 3.3.1. Corneal Fluorescein Staining

**Figure 2:**
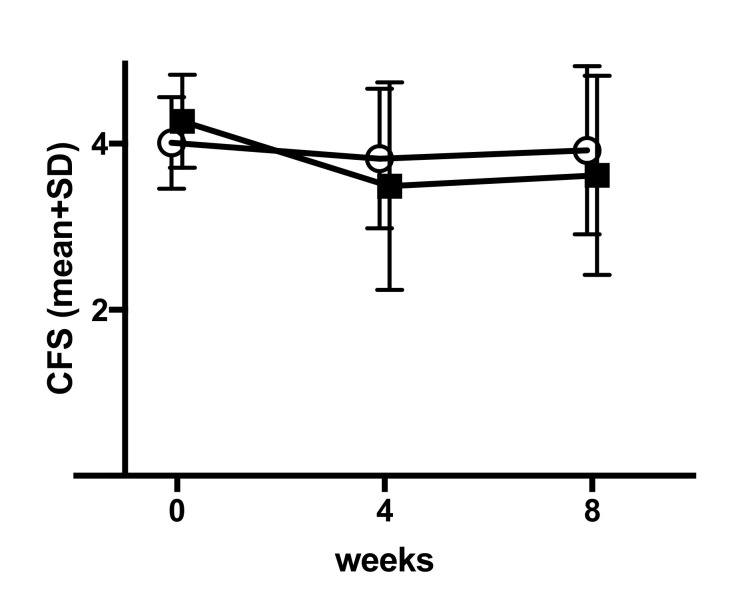
Mean (±SD) of corneal fluorescein staining (CFS) (central reading value, transformed into grade exact value) by group according time—PPS (*n* = 84). Open circles = Comfort Shield group, filled squares = control group.

The difference in corneal fluorescein staining (CFS) between baseline and week 8 determined by the masked reading center RC1 was the primary endpoint of the HYLAN M Study. The test method, electronic assessment, and calculation are described in Appendix D. Figure 2 and Table 3 describe the test results for the Comfort Shield group and the control group.

The changes from baseline to week 4 and to week 8 are documented in Table 4.

There was no significant (*p*-value < 0.05) difference between the two groups for the primary endpoint CFS, as documented in Table 5.

#### 3.3.2. Ocular Surface Disease Index

**Figure 3:**
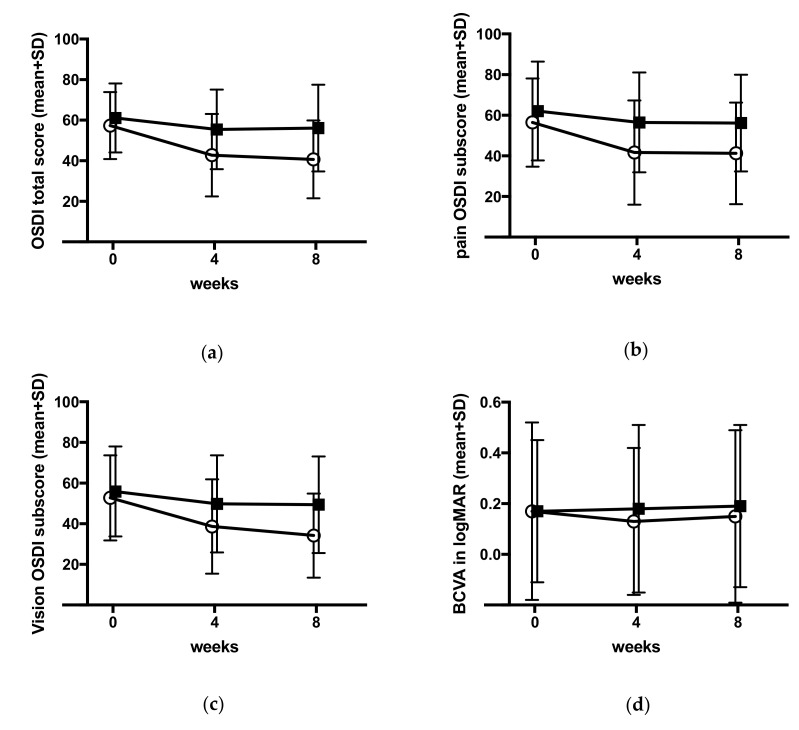
(**a**) Mean (±SD) OSDI total score; (**b**) mean (±SD) OSDI pain subscore; (**c**) mean (±SD) OSDI vision subscore; (**d**) mean (±SD) best corrected visual acuity (BCVA). Open circles = Comfort Shield group, filled squares = control group.

The key secondary endpoint of the HYLAN M study was the difference in ocular surface disease index (OSDI) between baseline and week 8 assessed by a questionnaire to be filled by the patients at the beginning of each visit. The Comfort Shield group had experienced at the end of the study (week 8 visit) significantly more relief from dry eye symptoms than the control group as documented in Figure 3a and Table 6, Table 7 and Table 8 (*p*-value 0.001).

The subscores for pain and visual stability-related symptoms were calculated and analyzed as described in the section on statistical analysis. The results are provided in Figure 3b,c and in Table 9 and Table 10. Both subscores OSDI_pain_ and OSDI_vision_ improved significantly in the Comfort Shield group as compared to the control group (*p*-values 0.002 and 0.003, respectively).

#### 3.3.3. Best Corrected Visual Acuity

The BCVA slightly improved after eight weeks of Comfort Shield treatment as compared to the control group (*p*-value 0.033). Details are provided in Figure 3d and Table 11.

#### 3.3.4. Other Secondary Endpoints

The secondary endpoints TBUT, Schirmer I, lid wiper epitheliopathy Korb score, Yamaguchi score, and tear film osmolarity are summarized in Table 12. No significant differences between the Comfort Shield group and control group were observed (all *p*-values > 0.05).

#### 3.3.5. Observation of the Subbasal Nerve Plexus by Confocal Microscopy

Confocal laser scanning microscopy was performed on 16 patients (eight patients each in the Comfort Shield group and in the control group) at four out of 11 study centers. Images of the subbasal nerve plexus were taken at baseline and week 8 and assessed at RC2. There was a significant increase of total nerve fiber length in the Comfort Shield group (51% growth; *p*-value 0.030), whereas in the control group, the total subbasal corneal nerve fiber length did not significantly change from baseline to week 8. Detailed results will be subject to a separate publication.

#### 3.3.6. Dropping Frequency

The patients were instructed to use their lubricant eye drops whenever ocular discomfort occured. They recorded the dropping daily. The average dropping frequency was not significantly different in the two treatment arms. By the time of inclusion into the study, the patients reported using 7.6 (minimum: 2; maximum: 36) artificial tear drops or autologous serum eye drops per day in the control group, and 8.2 (minimum: 3; maximum: 20) in the Comfort Shield Group. During week 8 of the study, the average dropping frequency was 6.5 (minimum: 1; maximum: 24.6) per day in the control group and 7.1 (minimum: 2; maximum: 23.8) in the Comfort Shield group.

#### 3.3.7. Influence of Climate on CFS and OSDI

In order to investigate whether or not climate has a significant impact on the study results, the primary endpoint CFS and the key secondary endpoint OSDI were analyzed separately for the nine study centers located in Europe and the two study centers in Riyadh in the desert region of Saudi Arabia. There were no significant differences between these two subgroups. The complete results are presented in Appendix G, Table A5, Table A6, Table A7 and Table A8.

### 3.4. Safety Results

The assessment of safety results refers to the safety set (SS), i.e., all patient that had at least once received eye drops (*n* = 121).

The average intraocular pressure in both study arms at baseline and week 8 was 14 mmHg. All values were between 8 and 23 mmHg. There were no patients suspect of uncontrolled glaucoma or ocular hypertension.

Of the Comfort Shield group, one patient discontinued the participation in the study after one week because the dry eye symptoms had worsened. Two patients reported during the week 4 visit about blurred vision for 10 min after the instillation of Comfort Shield eye drops, but they wanted to continue to participate in the study. One patient reported during the week 4 visit about persistent redness but wanted to continue to participate in the study. One patient reported during the week 4 visit about burning sensation, but they wanted to continue to participate in the study. One patient reported during the week 4 visit an episode of three days of red, painful, itching left eye, but they wanted to continue to participate in the study.

Of the control group, three patients had experienced not device-related adverse events between the week 4 and week 8 visits. One patient had nausea for two days, one patient had a mild viral conjunctivitis, and one patient had to be admitted to the hospital with cervical pain and was treated for six days with analgesics.

## 4. Discussion

The population of this study shows the typical predominance of age and female gender in dry eye disease, as the majority of patients was older than 45 years (90.4%, respectively, 65 years (33.3%) and most patients were female (82.1%) [54]. There was no significant difference between the verum and the control group.

The design of clinical trials on dry eye disease needs to consider symptoms, namely ocular discomfort and visual disturbance, as well as signs, such as tear film instability, damage of the ocular surface, increased tear osmolarity, and inflammation of the ocular surface [55]. International regulatory agencies rely on ocular surface staining as a primary endpoint for new drug approvals [56]. The ODISSEY European Consensus Group recommended CFS as the primary sign for severity of DED [39]. For this reason, the HYLAN M study used CFS as the primary endpoint of the study and standardized the test method as well as the objective assessment of staining as far as reasonably possible. Other dry eye signs, such as tear osmolarity, TBUT, Schirmer I, lid wiper epitheliopathy, and position of the mucocutaneous junction at the lid rim were chosen as secondary endpoints, having in mind the well-known poor correlation between symptoms and signs in DED. As corneal nerve damage has in recent years been recognized as an important pathomechanism in severe ocular surface disease, the assessment of the subbasal nerve plexus using confocal laser scanning microscopy had been included as an additional optional test in the study design. OSDI was chosen as the key secondary endpoint for the assessment of dry eye symptoms as it is widely used and easy to interpret.

The HYLAN M study did not find a statistically significant difference between the Comfort Shield group and the control group at the week 8 visit for the primary endpoint CFS or any of the following secondary endpoints: TBUT, Schirmer I, lid wiper epitheliopathy, and tear osmolarity. This emphasizes that in patients with severe dry eyes, a change from the therapy with individualized lubricant eye drops to HMWHA eye drops does not result in a worsening of dry eye signs.

The primary endpoint of the HYLAM M study, CFS, did not show any significant difference between the two study arms. The value of CFS as an absolute number to judge the improvement or deterioration of the corneal surface condition has been questioned. As it is known that the difference in grading between different investigators may limit the sensitivity of detectable changes in CFS over time, the CFS test method within the HYLAN M study had been highly standardized, and a reading center performing electronic assessment of CFS images has been involved (see Appendix D). The well-known, possibly even physiological variation of CFS between measurements has been supported by the present study. Moreover, it is known that CFS is sensitive to effects of quenching and pooling, which may affect the repeatability and accuracy of measurement. A post hoc analysis of the control group demonstrated that there is a significant fluctuation in CFS over time even in the best treated patients under stable optimum treatment (see Appendix H). This variation emphasizes the difficulty of judging the ocular surface condition from surface staining intensity. Similar fluctuations such as the one experienced for CFS are known for other dry eye signs [17,52,57,58,59,60,61,62,63]. Originally enthusiastically welcomed as a highly reliable parameter for DED and used as a major decision maker with respect to the severity of DED, the diagnostic significance of tear osmolarity determined in the lower tear meniscus has recently been questioned [64]. The average osmolarity of 298 mOsm/L found in the HYLAN M study for patients suffering from severe DED is not hyperosmotic, as expected for severe DED.

OSDI was the key secondary endpoint of the HYLAN M study. The OSDI questionnaire is one of the most commonly used tests to assess dry eye symptoms [42]. The OSDI score was assessed at the baseline visit, after four weeks, and after eight weeks. Whereas, in the control group, the OSDI score slightly improved in the first four weeks, which was presumably due to better compliance of the patients with their treatment regimen, but it did not further improve beyond the four-week study participation. Contrarily, the OSDI score significantly improved under Comfort Shield treatment in the first four weeks and continued to improve in the second four-week period (see Figure 3a). Such improvement applies also to the subscore for discomfort and pain, as well as for the subscore for visual instability (see Figure 3b,c). After eight weeks treatment, the difference between the Comfort Shield group and the control group were for the total OSDI score 13.5 (*p*-value 0.001), for the pain subscore 14.5 (*p*-value 0.002), and for the vision subscore 14.0 (*p*-value 0.003). This unexpectedly great improvement in dry eye symptoms under the treatment with HMWHA eye drops deserves further investigation. The Asia Dry Eye Society recently concluded that subjective severity (symptoms) could be used as a marker for therapeutic efficacy in dry eye treatment [9].

The improvement of the symptoms of visual stability in the HYLAN M study was reflected by a minor but significant improvement of BCVA. Whereas, BCVA determines the best visual acuity within a certain period of time, functional visual acuity continuously determines visual acuity and, therefore, better reflects the subjective stability of vision [65,66,67]. Therefore, in future clinical studies on dry eye disease, functional visual acuity rather than BCVA might be used as an endpoint.

As an optional test within the HYLAN M study, the subbasal nerve plexus was analyzed in a subgroup of 16 patients. There was a significant increase of total nerve fiber length in the Comfort Shield group as compared to the control group after eight weeks of treatment. This observation correlates well with the significant improvement of pain symptoms. The fact that at the same time there was no significant change of other dry eye signs suggests that the observed therapeutic effect cannot be attributed to a physical effect of the eye drops such as hydration or lubrication, but it is likely to result from a pharmacological effect downregulating ocular inflammation and supporting corneal nerve recovery.

## 5. Conclusions

In an international multicenter randomized clinical study on patients suffering from severe dry eye disease, 0.15% high molecular weight hyaluronan (HMWHA) eye drops have been compared with lubricant eye drops individually selected as optimum therapy. HMWHA eye drops have shown superior potential to significantly ameliorate symptoms including discomfort and pain, as well as visual instability without affecting dry eye signs.

## Figures and Tables

**Table 1:** Diagnostic testing schedule with optional tests in round brackets.

Test	Baseline	Week 4	Week 8
OSDI	X	X	X
dropping frequency	X	X	X
BCVA	X	X	X
CFS	X	X	X
TBUT	X	X	X
Schirmer 1	X		X
Tear osmolarity	X		X
IOP	X		X
LWE Korb score	(X)		(X)
Yamaguchi score	(X)		(X)
Confocal microscopy	(X)		(X)

**Table 2:** Climate zone and socio-demographic characteristics according to the treatment arm– per-protocol set (PPS) population (*n* = 84).

		Comfort Shield (*n* = 44)	Control (*n* = 40)	Total (*n* = 84)
climate zone	*n*	44	40	84
	Desert	6 (13.6)	7 (17.5)	13 (15.5)
	Europe	38 (86.4)	33 (82.5)	71 (84.5)
	missing	0	0	0
age (years)	*n*	44	40	84
	mean (sd)	57.66 (14.39)	59.45 (12.48)	58.51 (13.46)
	median (iqr)	61.5 (50.75, 65.25)	60 (51.5, 69.0)	61 (50.75, 67.00)
	min, max	26, 81	27, 84	26, 84
	missing	0	0	0
age (years)	*n*	44	40	84
	<40	6 (13.6)	2 (5.0)	8 (9.5)
	[40–65[	25 (56.8)	23 (57.5)	48 (57.1)
	≥65	13 (29.5)	15 (37.5)	28 (33.3)
	missing	0	0	0
sex *n* (%)	*n*	44	40	84
	female	35 (79.5)	34 (85.0)	69 (82.1)
	male	9 (20.5)	6 (15.0)	15 (17.9)
	missing	0	0	0
ethnicity *n* (%)	*n*	44	40	84
	Arabian	7 (15.9)	8 (20.0)	15 (17.9)
	Caucasian	36 (81.8)	31 (77.5)	67 (79.8)
	other	1 (2.3)	1 (2.5)	2 (2.4)
	missing	0	0	0

Abbreviations: sd = standard deviation; iqr = interquartile range; min = minimum; max = maximum [53].

**Table 3:** CFS: Value at baseline and at each post-baseline visit—PPS (*n* = 84)—Descriptive analysis, by group.

		Comfort Shield (*n* = 44)	Control (*n* = 40)	Total (*n* = 84)
value at baseline	*n*	44	40	84
	mean (sd)	4.01 (0.55)	4.27 (0.56)	4.13 (0.57)
	median (iqr)	3.92 (3.55, 4.37)	4.39 (3.72, 4.67)	4.18 (3.6, 4.6)
	min, max	3.0, 5.0	3.12, 5.00	3.0, 5.0
	missing	0	0	0
value at week 4	*n*	41	38	
	mean (sd)	3.82 (0.84)	3.49 (1.25)	
	median (iqr)	4 (3.26, 4.46)	3.8 (2.82, 4.36)	
	min, max	1.35, 5.0	0.0, 5.0	
	missing	3	2	
value at week 8	*n*	41	38	
	mean (sd)	3.91 (1.04)	3.62 (1.2)	
	median (iqr)	4.31 (3.38, 4.68)	3.82 (3.27, 4.43)	
	min, max	0.0, 5.0	0.0, 5.0	
	missing	3	2	

**Table 4:** CFS: Value at baseline and change from baseline to each post-baseline visit—PPS (*n* = 84)—Descriptive analysis, by group.

		Comfort Shield (*n* = 44)	Control (*n* = 40)	Total (*n* = 84)
baseline	*n*	44	40	84
	mean (sd)	4.01 (0.55)	4.27 (0.56)	4.13 (0.57)
	median (iqr)	3.92 (3.55, 4.37)	4.39 (3.72, 4.67)	4.18 (3.6, 4.6)
	min,max	3.0, 5.0	3.12, 5.00	3.0, 5.0
	missing	0	0	0
change from baseline to week 4	*n*	41	38	
	mean (sd)	−0.22 (0.76)	−0.76 (1.02)	
	median (iqr)	−0.06 (−0.70, 0.28)	−0.68 (−1.02, −0.20)	
	min,max	−2.54, 1.13	−4.41, 0.90	
	missing	3	2	
change from baseline to week 8	*n*	41	38	
	mean (sd)	−0.13 (1.08)	−0.63 (1.05)	
	median (iqr)	−0.07 (−0.57, 0.58)	−0.33 (−0.99, 0.10)	
	min,max	−4.73, 1.45	−3.60, 0.84	
	missing	3	2	

**Table 5:** CFS: multivariate analysis on change from baseline to week 8—mixed-effects model for repeated measures—PPS population (*n* = 84). *n* used = 79.

Parameter	Comparison	E^(1)^	CI 95% Low	CI 95% High	*p*-Value ^(2)^
Change from baseline to week 8	Control vs. Comfort Shield	−0.411	−0.865	0.043	0.075

^(1)^ Estimate (E) and associated 95% two-sided confidence interval (CI) of the difference between treatment group adjusted means: mixed-effects model for repeated measures (MMRM) with the fixed, categorical effects of treatment, visit, and treatment-by-visit interaction, the random categorical effect of center, as well as the continuous, fixed covariates of baseline and baseline-by-visit interaction. A positive estimate of the difference between treatment group adjusted means is in favour of the Comfort Shield, a negative ones in disfavor of the Comfort Shield. ^(2)^ two-sided *p*-value associated with the test of treatment effect.

**Table 6:** Ocular surface disease index (OSDI): value at baseline and at each post-baseline visit—PPS (*n* = 84)—descriptive analysis, by group.

		Comfort Shield (n = 44)	Control (*n* = 40)	Total (*n* = 84)
value at baseline	*n*	44	40	84
	mean (sd)	57.41 (16.5)	61.13 (16.99)	59.18 (16.74)
	median (iqr)	54.55 (43.61, 68.75)	61.8 (51.56, 75.00)	57.91 (43.75, 70.45)
	min, max	34.09, 91.67	34.09, 95.45	34.09, 95.45
	missing	0	0	0
value at week 4	*n*	44	40	
	mean (sd)	42.82 (20.34)	55.49 (19.64)	
	median (iqr)	39.2 (29.79, 56.96)	54.03 (38.07, 68.50)	
	min, max	7.14, 85.42	22.22, 95.45	
	missing	0	0	
value at week 8	*n*	44	40	
	mean (sd)	40.7 (19.18)	56.16 (21.39)	
	median (iqr)	40.91 (28.13, 52.81)	57.91 (36.11, 75.00)	
	min, max	0.0, 87.5	9.09, 93.18	
	missing	0	0	

**Table 7:** OSDI: value at baseline and change from baseline to each post-baseline visit—PPS (*n* = 84)—Descriptive analysis, by group.

		Comfort Shield (*n* = 44)	Control (*n* = 40)	Total (*n* = 84)
value at baseline	*n*	44	40	84
	mean (sd)	57.41 (16.5)	61.13 (16.99)	59.18 (16.74)
	median (iqr)	54.55 (43.61, 68.75)	61.8 (51.56, 75.00)	57.91 (43.75, 70.45)
	min, max	34.09, 91.67	34.09, 95.45	34.09, 95.45
	missing	0	0	0
change from baseline to week 4	*n*	44	40	
	mean (sd)	−14.6 (20.71)	−5.63 (12.66)	
	median (iqr)	−10.21 (−25.80, −2.09)	−3.98 (−12.5, 0.0)	
	min, max	−70.83, 27.27	−37.92, 25.76	
	missing	0	0	
change from baseline to week 8	*n*	44	40	
	mean (sd)	−16.71 (22.25)	−4.96 (16.95)	
	median (iqr)	−13.41 (−29.66, −0.68)	−3.82 (−13.02, 6.99)	
	min, max	−84.09, 27.08	−43.06, 40.91	
	missing	0	0	

**Table 8:** OSDI: Multivariate analysis on change from baseline to week 8—mixed-effects model for repeated measures—PPS population (*n* = 84)—*n* used = 84.

Parameter	Comparison	E ^(1)^	CI 95% Low	CI 95% High	*p*-Value ^(2)^
Change from baseline to week 8	Control vs. Comfort Shield	13.511	5.586	21.437	0.001

^(1)^ Estimate (E) and associated 95% two-sided confidence interval (CI) of the difference between treatment group adjusted means: MMRM with the fixed, categorical effects of treatment, visit, and treatment-by-visit interaction, the random categorical effect of center, as well as the continuous, fixed covariates of baseline and baseline-by-visit interaction. A positive estimate of the difference between treatment group adjusted means is in favor of the Comfort Shield, a negative one in disfavor of the Comfort Shield. ^(2)^ two-sided *p*-value associated with the test of treatment effect.

**Table 9:** Pain OSDI subscore: multivariate analysis on change from baseline to week 8—mixed-effects model for repeated measures—PPS population (*n* = 84)—*n* used = 84.

Parameter	Comparison	E ^(1)^	95% CI Low ^(1)^	95% CI High ^(1)^	*p*-Value ^(2)^
Change from baseline to week 8	Comfort Shield vs. Control	14.503	5.517	23.49	0.002

^(1)^ Estimate (E) and associated 95% two-sided confidence interval (CI) of the difference between treatment group adjusted means: MMRM with the fixed, categorical effects of treatment, visit, and treatment-by-visit interaction, the random categorical effect of center, as well as the continuous, fixed covariates of baseline and baseline-by-visit interaction. A positive estimate of the difference between treatment group adjusted means is in favour of the Comfort Shield, a negative one in disfavour of the Comfort Shield. ^(2)^ two-sided *p*-value associated with the test of treatment effect.

**Table 10:** Vision OSDI subscore: Multivariate analysis on change from baseline to week 8—mixed-effects model for repeated measures—PPS population (*n* = 84)–*n* used = 84.

Parameter	Comparison	E^(1)^	95% CI Low^(1)^	95% CI High^(1)^	*p*-Value^(2)^
Change from baseline to week 8	Comfort Shield vs. Control	13.999	5.011	22.986	0.003

^(1)^ Estimate (E) and associated 95% two-sided confidence interval (CI) of the difference between treatment group adjusted means: MMRM with the fixed, categorical effects of treatment, visit, and treatment-by-visit interaction, the random categorical effect of center, as well as the continuous, fixed covariates of baseline and baseline-by-visit interaction. A positive estimate of the difference between treatment group adjusted means is in favour of the Comfort Shield, a negative one in disfavor of the Comfort Shield. ^(2)^ two-sided *p*-value associated with the test of treatment effect.

**Table 11:** BCVA: value at baseline and change from baseline to each post-baseline visit—PPS (*n* = 84)—descriptive analysis, by treatment arm.

		Comfort Shield (*n* = 44)	Control (*n* = 40)	Total (*n* = 84)	*p*-Value
baseline	*n*	44	40	84	
	mean (sd)	0.17 (0.35)	0.17 (0.28)	0.17 (0.31)	
	median (iqr)	0 (0.0, 0.2)	0.1 (0.00, 0.22)	0 (0.0, 0.2)	
	min, max	−0.1, 1.5	−0.2, 1.3	−0.2, 1.5	
	missing	0	0	0	
change from baseline to week 4	*n*	41	39		
	mean (sd)	0 (0.11)	0.02 (0.11)		
	median (iqr)	0 (0, 0)	0 (0.00, 0.05)		
	min, max	−0.4, 0.3	−0.2, 0.5		
	missing	3	1		
change from baseline to week 8	*n*	44	40		0.033
	mean (sd)	−0.02 (0.14)	0.02 (0.1)		
	median (iqr)	0 (−0.1, 0.0)	0 (0.0, 0.1)		
	min, max	−0.4, 0.6	−0.2, 0.3		
	missing	0	0		

**Table 12:** Values at baseline and change from baseline to week 8 for tear film break-up time (TBUT), Schirmer I, lid wiper epitheliopathy (LWE) Korb score, Yamaguchi score, and tear osmolarity—PPS (*n* = 84).

	Comfort Shield Group *n* = 44		Control Group *n* = 40		*p*-Value
	Baseline Mean (SD)	Change at Week 8 Mean (SD)	Baseline Mean (SD)	Change at Week 8 Mean (SD)	Change from Baseline to Week 8
TBUT (s)	*n* = 44 2.90 (1.87)	*n* = 42 0.66 (2.32)	*n* = 40 2.76 (1.44)	*n* = 40 0.24 (1.47)	0.468
Schirmer I (mm/5 min)	*n* = 44 5.19 (5.99)	*n* = 43 −0.43 (4.72)	*n* = 40 6.50 (7.52)	*n* = 40 0.55 (4.61)	0.343
LWE Korb score	*n* = 37 1.22 (1.00)	*n* = 37 −0.19 (0.92)	*n* = 37 0.91 (0.96)	*n* = 37 0.12 (0.71)	0.153
Yamaguchi score	*n* = 37 5.05 (2.33)	*n* = 37 −0.14 (2.42)	*n* = 37 4.14 (2.41)	*n* = 37 0.19 (1.97)	0.498
tear osmolarity * (mOsm/L)	*n* = 40 297.12 (14.47)	*n* = 37 2.11 (14.54)	*n* = 37 299.16 (12.11)	*n* = 35 0.94 (17.59)	0.294

* See Appendix F for details of statistical handling of measurement values below the detection limit of the TearLab test instrument.

**Figure A1:**
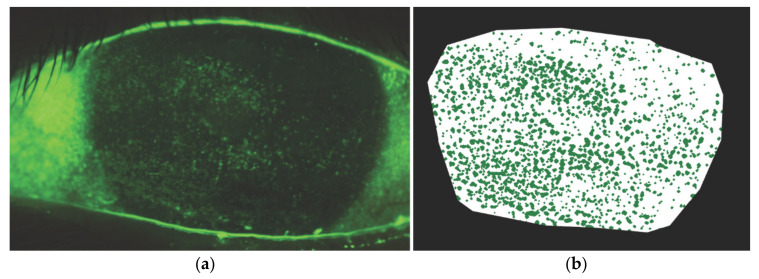
(**a**) CFS image as uploaded by the trial sites to the reading center; (**b**) CFS image after manually segmenting the cornea and application of a threshold-based segmentation of the CFS-positive lesions. The final readout is the percentage of total green pixels divided by the total pixels of the cornea area.

**Figure A2:**
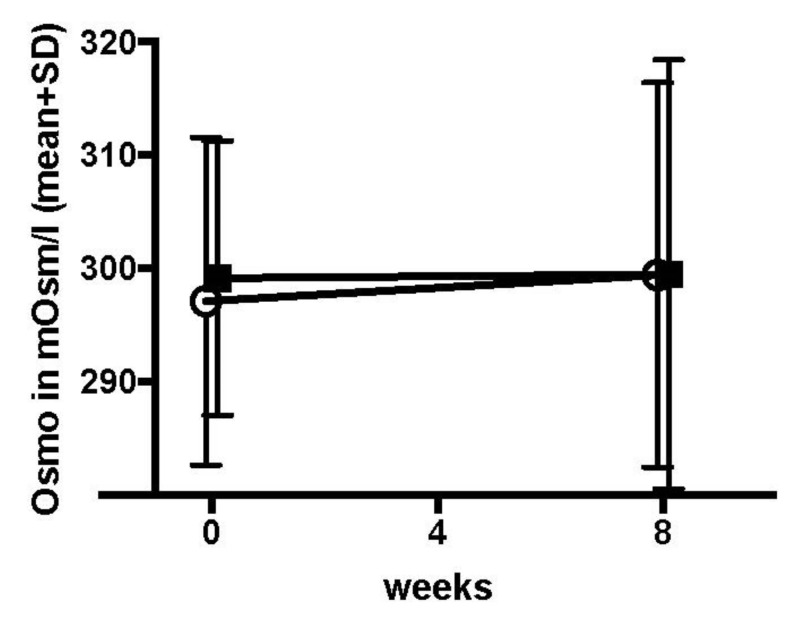
Mean (±SD) of osmolarity by treatment arm according time—PPS (*n* = 84). Open circles = Comfort Shield group, filled squares = control group.

**Figure A3:**
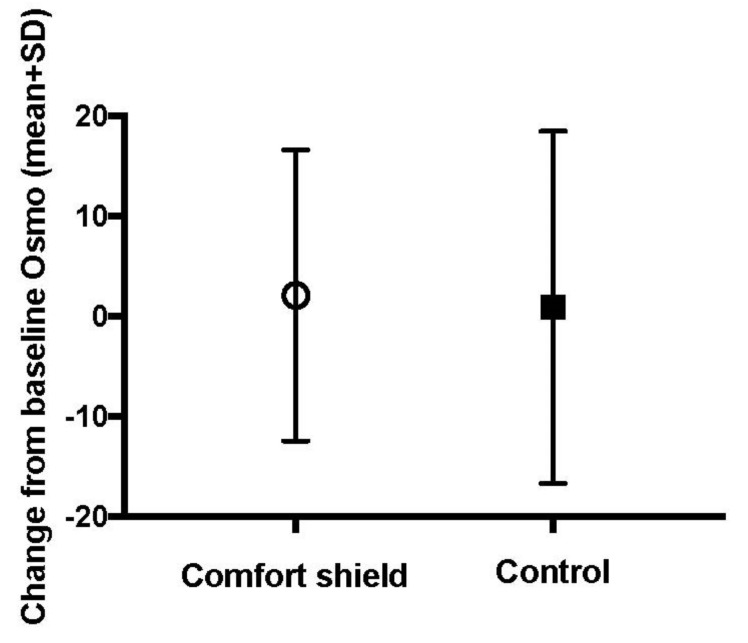
Mean (±SD) change from baseline to week 8 (week 8–baseline) of osmolarity by group—PPS (*n* = 84). Open circle = Comfort Shield group, filled square = control group.

## Appendix A. Study Centers, Administrative Structure of the Study, and Author Contributions

Eleven study centers in eight countries with different climate zones and different ethnicities participated in the HYLAN M Study:

(1) Centre Hospitalier National d’Ophtalmologie—Quinze-Vingts, Paris, France, principle investigator (PI) Christophe Baudouin
(2) St. Erik Eye Hospital, Stockholm, Sweden, PI Gysbert-Botho van Setten
(3) Department of Ophthalmology, University Medical Center Rostock, Rostock, Germany, PI Ria Beck
(4) Department of Ophthalmology, Medical University Graz, Graz, Austria, PI Jutta Horwath-Winter
(5) Department of Ophthalmology, University Clinic Salzburg, Paracelsus Medical University, Salzburg, Austria, PI Herbert A. Reitsamer
(6) Department of Ophthalmology, Saarland University Medical Center, Homburg/Saar, Germany, PI Berthold Seitz
(7) KKESH—King Khaled Eye Specialist Hospital, Riyadh, Saudi Arabia, PI Osama Al-Sheikh
(8) Marmara University Pendik Training and Research Hospital, Marmara University Medical School, Department of Ophthalmology, Istanbul, Turkey, PI Ebru Toker
(9) Department of Ophthalmology, PSMMC Prince Sultan Military Medical City, MSD Medical Services Department, MODA Ministry of Defense and Aviation, Riyadh, Saudi Arabia, PI Sultan Al-Zaaidi
(10) Universidad Complutense de Madrid, Hospital Clinico San Carlos, Departamentos de Oftalmologia, Madrid, Spain, PI Jose M. Benitez-del-Castillo
(11) Ocular Surface & Dry Eye Center, Ospedale L. Sacco, University of Milan, Milan, Italy, PI Stefano Barabino

The HYLAN M Study was designed in accordance with the international standard ISO 14155:2012 by the sponsor CORONIS GmbH (Munich, Germany) in close cooperation with the PIs Christophe Baudouin, Gysbert van Setten, and Jutta Horwath-Winter, the representatives of the reading centers, Daniel Böhringer for RC1 and Oliver Stachs for RC2, the biostatistician Sébastian Marque (IQVIA, France), and the randomization center (C2R, Paris, France). CORONIS is operating under a quality management system including clinical research, which was certified according to the international standard 13485:2016 and annually supervised by the European notified body mdc medical device certification GmbH (Stuttgart, Germany).

The representative of the sponsor, Wolfgang G.K. Müller-Lierheim, had overall responsibility as study director (SD). In obtaining local ethics committee approval, registering the study with national authorities, and monitoring the study, he was supported by

- IPR—International Pharmaceutical Consultancy, Paris, France
- OPIS, Desio, Italy
- Monitor Medikal Araştırma ve Danışmanlık Tic. Ltd. Şti., Istanbul, Turkey
- KKESH Research Department, Riyadh, Kingdom of Saudi Arabia.

The medical scientific management of the HYLAN M Study was under the overall responsibility of the coordinating investigator (CI) Gysbert-Botho van Setten, who was supported by Jutta Horwath-Winter with respect to the lissamine green staining procedure and diagnostic judgement, and Oliver Stachs regarding confocal microscopy.

The HYLAN M Study used detailed work instructions including the diagnostic procedures corneal fluorescein staining (see Appendix D), tear film break-up time, lissamine green staining (including assessment of lid wiper epitheliopathy and mucocutaneous junction), tear film osmolarity using the TearLab osmolarity system (TearLab Corporation, San Diego, CA, USA), and taking and evaluating confocal microscopy images of the subbasal nerve plexus using the Heidelberg Retina Tomograph HRT in combination with the Rostock Cornea Module RCM (Heidelberg Engineering GmbH, Heidelberg, Germany).

Paper case report forms (CRF) were used by the study centers throughout the study and were archived by each study center for a minimum of 10 years beyond the completion of the HYLAN M study. The contents of the case report forms were anonymously transferred to electronic case report forms (eCRF) using a web-based system allowing electronic transfer to the HYLAN M study database hosted at the University Eye Hospital in Freiburg, Germany. For monitoring purposes, the SD had online access to the HYLAN M database. Printouts of the eCRFs were used to assure the correct and complete data transfer from the paper CRFs to the eCRFs during regular monitoring visits of the study centers. At the closure visits of each study center, printouts of all eCRFs were signed by the responsible PI and provided to the sponsor who scanned and electronically archived the signed eCRFs. After the closing the last study center and monitoring the reading centers and the host of the study database, the database was locked on 26 March 2020, and exported for statistical analysis.

The company C2R (Paris, France) under the responsibility of the data managers Pascale Croix and Mélinda Ezzedine provided the electronic block randomization of patients of the HYLAN M study in two equally sized study arms A (control group) and B (Comfort Shield group = verum group). The investigators were kept unaware of block randomization and the block size of four. For the randomization, the module CSRandomization of the ENNOV CLINICAL^®^ software (version 7.5) was used. The clinical data management system had been developed and validated to meet all regulatory requirements of data management and create a non-modifiable audit trail. The study centers had password-protected secure CS Online internet interface access to the randomization tool. After entering the patient number, the system automatically created the result of randomization. Then, the study centers printed the patient number and result of randomization and filed this together with their CRFs to be controlled during monitoring visits.

The HYLAN M Study used two masked reading centers for the analysis of digital images provided by the study centers. The cornea reading center located at the Eye Center, University Hospital Freiburg, Germany, in the text referred to as RC1, analyzed the corneal fluorescein staining (see Appendix D). Oliver Stachs (Department of Ophthalmology, University Medical Center, Rostock, Germany), in the text referred to as RC2, analyzed the subbasal nerve fibers in confocal microscopy images, which were taken as an additional optional diagnostic test at the baseline and week 8 visits of the HYLAN M study (the results of this subgroup analysis will be published separately).

The statistical analysis plan (SAP) for the HYLAN M Study was developed by IQVIA, Bordeaux, France in cooperation with the SD. IQVIA performed the statistical analysis by using the software R version 3.5.3 (The R Foundation for Statistical Computing, Vienna, Austria) after database lock and provided the statistical analysis report (SAR) of the HYLAN M study. Upon request, the sponsor of the HYLAN M study will provide a copy of the SAR.

The statistical analysis of the results from the optional assessment of the subbasal nerve plexus by confocal laser scanning microscopy was performed by RC2.

The manuscript of this report was prepared by Gysbert-Botho van Setten, Jutta Horwath-Winter, Daniel Böhringer, Oliver Stachs, and Wolfgang G.K. Müller-Lierheim.

## Appendix B. Ethics Committee Approval, Compliance with the Declaration of Helsinki, and Registration of the Study

The HYLAN M study was approved by ethics committees in all eight countries where study centers were located:

- Austria: Medical University Graz, Ethic Committee, reg. no. 28-458 ex 15/16
- France: Comite de protection des Persones Ile de France V, reg. no. 16138
- Germany: Ärztekammer des Saarlandes, Ethik-Kommission, reg. no. 176/16
- Italy: Comitato Etico Milano Area 1, protocol no. 47068/2018
- Kingdom of Saudi Arabia: King Khaled Eye Specialist Hospital, Research Department, reference RSCH/665/5957-16 and Prince Sultan Military Medical City, Research Ethics Committee, reg. no. HAP-01-R-015
- Spain: CEIC Hospital Clinico San Carlos, reg. no. 18/016-R_P
- Sweden: EPN Regionala etikprövningsnämnden I Stockholm protocol 19 October 2016
- Turkey: Marmara University Hospital, Klinik Arastirmalar Etik Kurulu, form 2013-KAEK-60

All subjects gave their informed consent for inclusion before they participated in the HYLAN M Study. The study was conducted in accordance with the Declaration of Helsinki.

The HYLAN M study was registered on the database of the European Commission for medical devices EUDAMED under the registration number CIV-16-06-015964. Moreover, the study was registered with national competent authorities in Austria, France, Germany, Italy, Sweden, and Turkey.

## Appendix C. Investigational Device

The control group of the HYLAN M study continued to use the lubricant eye drops which the individual participant had been using by the time of inclusion. In the verum group (Comfort Shield group), the lubricant eye drops which the individual participant had been using by the time of inclusion were replaced by Comfort Shield^®^ eye drops (i.com medical GmbH, Munich, Germany). Comfort Shield^®^ eye drops were available to the participants in two dosage forms with identical preservative-free composition, as Comfort Shield^®^ SD in boxes containing 15 monodoses each, and as Comfort Shield^®^ MDS in 10 mL bottles. Comfort Shield^®^ contains 0.15% high molecular weight hyaluronan (Hylan A; intrinsic viscosity 2.9 m^3^/kg) dissolved in isotonic saline solution with 1.20 mmol/L phosphate buffer.

Comfort Shield^®^ eye drops are approved in Europe as Class IIb medical device and were used within the HYLAN M study in accordance with the labeling.

## Appendix D. Corneal Fluorescein Staining and Electronic Analysis Method

Sterile, preservative-free 0.5% sodium fluorescein solution in 0.4 mL monodose containers (FLUORESCEINE FAURE 0.5 PER CENT ophthalmic solution in unit dose, SERB SAS, Paris, France) and Eppendorf Reference 2 pipettes with 10 µl fixed volume (Eppendorf AG, Hamburg, Germany) were provided by the sponsor to each study center.

Then, 10 µl fluorescein solution was released in the cul-de-sac of the patient’s eye without touching the ocular surface. The patient blinked five times to evenly distribute the fluorescein on the ocular surface. After 20 to 120 s, the corneal and conjunctival fluorescein staining was judged visually at 16× magnification using the slitlamp with “cobalt” blue light illumination and a yellow barrier filter in the observation beam and graded using the Oxford score [41]. Digital images are taken. The images are electronically transferred to the reading center RC1 with the file names containing the patient number, visit, and eye (OD = right eye or OS = left eye).

During the qualification of the study centers, each study center submitted images of corneas with fluorescein staining together with their visual Oxford grading of staining to ensure and verify that RC1 will correctly assess the staining grade. Then, the settings of slitlamp, illumination, filters, and camera were fixed and kept constant throughout the HYLAN M study.

RC1 analyzed the staining in two steps. Figure A1a shows a typical image that is uploaded to the reading center. First, the region of interest comprising the cornea to be analyzed is segmented manually. In the second step, the CSF-positive lesions are electronically segmented using a threshold-based image processing algorithm. Figure A1b shows the image after electronic processing. The final readout is the percentage of total green pixels divided by the total pixels of the cornea area. The parameters for the automated segmentation were carefully set up for each study center individually as part of the center certification procedure.

RC1 had empirically found that 2.00% staining correlates well with Oxford grade 3, and therefore, this was used as the inclusion criteria to be met at least by one eye of the patient. Patients with confluent staining were excluded because the staining cannot be electronically quantified.

The Oxford grading is based on 0.5 log units difference between grade 1 and grade 5 [41]. Therefore, for the transformation percentage of fluorescein staining into “continuous Oxford grades”, the following formula was used:

If staining < 0.2% → Grade = 0

If staining [0.2–20%] → Grade = 2.398 + 2 × log_10_(% staining)

If staining > 20% → Grade = 5.

## Appendix E. Medical History

**Table A1:** Tabular overview of the medical history according to the treatment arm—PPS population (*n* = 84).

		Comfort Shield (*n* = 44)	Control (*n* = 40)	Total (*n* = 84)
Rheumatoid disease *n* (%)	*n*	43	40	83
	no	21 (48.8)	20 (50.0)	41 (49.4)
	yes	22 (51.2)	20 (50.0)	42 (50.6)
	missing	1	0	1
Thyroid disease *n* (%)	*n*	43	40	83
	no	27 (62.8)	33 (82.5)	60 (72.3)
	yes	16 (37.2)	7 (17.5)	23 (27.7)
	missing	1	0	1
Trachoma *n* (%)	*n*	43	40	83
	no	43 (100.0)	40 (100.0)	83 (100.0)
	missing	1	0	1
Other disease *n* (%)	*n*	43	39	82
	no	23 (53.5)	17 (43.6)	40 (48.8)
	yes	20 (46.5)	22 (56.4)	42 (51.2)
	missing	1	1	2
History betablocker *n* (%)	*n*	43	40	83
	no	33 (76.7)	33 (82.5)	66 (79.5)
	yes	10 (23.3)	7 (17.5)	17 (20.5)
	missing	1	0	1
Antidepressants *n* (%)	*n*	43	40	83
	no	36 (83.7)	34 (85.0)	70 (84.3)
	yes	7 (16.3)	6 (15.0)	13 (15.7)
	missing	1	0	1
Other drugs *n* (%)	*n*	42	38	80
	no	20 (47.6)	11 (28.9)	31 (38.8)
	yes	22 (52.4)	27 (71.1)	49 (61.2)
	missing	2	2	4
History contact lenses *n* (%)	*n*	43	40	83
	no	42 (97.7)	40 (100.0)	82 (98.8)
	yes	1 (2.3)	0 (0.0)	1 (1.2)
	missing	1	0	1
Conjunctival injection *n* (%)	*n*	43	39	82
	no	25 (58.1)	20 (51.3)	45 (54.9)
	yes	18 (41.9)	19 (48.7)	37 (45.1)
	missing	1	1	2
Inflammation of lid rim *n* (%)	*n*	43	39	82
	no	30 (69.8)	28 (71.8)	58 (70.7)
	yes	13 (30.2)	11 (28.2)	24 (29.3)
	missing	1	1	2

Six patients of the Comfort Shield group and two patients of the control group had received autologous serum eye drops by the time of inclusion into the study. In the Comfort Shield group, the autologous serum therapy was substituted by Comfort Shield eye drops.

Fifteen (34.1%) of 44 patients in the Comfort Shield group and 15 (37.5%) of 40 patients in the control group received cyclosporine eye drops and continued their application throughout the study.

Twenty-five (56.8%) of 44 patients in the Comfort Shield group and 23 (57.5%) of 40 patients in the control group were using hyaluronan containing artificial tears by the time of inclusion into the HYLAN M study.

## Appendix F. Tear Osmolarity Test Results

**Table A2:** Osmolarity: value in mOsm/L at baseline and at week 8—PPS (*n* = 84)—Descriptive analysis, by treatment arm.

		Comfort Shield (*n* = 44)	Control (*n* = 40)	Total (*n* = 84)
value at baseline	*n*	40	37	77
	mean (sd)	297.12 (14.47)	299.16 (12.11)	298.1 (13.34)
	median (iqr)	295.5 (287.75, 304.25)	299 (292, 307)	298 (289, 306)
	min, max	277, 337	275, 336	275, 337
	* <275 mOsm/L	2	2	4
	missing	2	1	3
value at week 8	*n*	40	37	
	mean (sd)	299.43 (16.99)	299.49 (18.94)	
	median (iqr)	300 (286, 308)	299 (291, 304)	
	min, max	275, 353	276, 395	
	* <275 mOsm/L	1	3	
	missing	3	0	

* values below detection limit (<275 mOsm/L) were not included in the statistical analysis.

**Table A3:** Osmolarity: Value in mOsm/L at baseline and at week 8—PPS (*n* = 84)–Descriptive analysis, by treatment arm.

		Comfort Shield (*n* = 44)	Control (*n* = 40)	Total (*n* = 84)
value at baseline	*n*	42	39	81
	<275 mOsm/L	2 (4.8)	2 (5.1)	4 (4.9)
	≥275 mOsm/L	40 (95.2)	37 (94.9)	77 (95.1)
	missing	2	1	3
value at week 8	*n*	41	40	
	<275 mOsm/L	1 (2.4)	3 (7.5)	
	* ≥275 mOsm/L	40 (97.6)	37 (92.5)	
	missing	3	0	

* values below detection limit (<275 mOsm/L) were not included in the statistical analysis.

**Table A4:** Osmolarity: value in mOsm/L at baseline and change from baseline to week 8–PPS (*n* = 84)–Descriptive analysis, by treatment arm.

		Comfort Shield (*n* = 44)	Control (*n* = 40)	Total (*n* = 84)	*p*-Value
value at baseline	*n*	40	37	77	
	mean (sd)	297.12 (14.47)	299.16 (12.11)	298.1 (13.34)	
	median (iqr)	295.5 (287.75, 304.25)	299 (292, 307)	298 (289, 306)	
	min, max	277, 337	275, 336	275, 337	
	* <275 mOsm/L	2	2	4	
	missing	2	1	3	
change from baseline to week 8	*n*	37	35		0.294
	mean (sd)	2.11 (14.54)	0.94 (17.59)		
	median (iqr)	2 (−6, 12)	−1 (−7.0, 7.5)		
	min, max	−29, 27	−30, 80		
	* <275 mOsm/L	1	3		
	** missing	6	2		

* No missing value but at least one value below detection limit (<275 mOsm/L). ** At least one missing value at baseline or Week 8.

## Appendix G. Influence of Climate on CFS and OSDI

The primary endpoint CFS and the key secondary endpoint were separately assessed for two subgroups: “Europe” (patients from all European study centers, including Istanbul) and “Desert” (patients from the two study centers in Riyadh, Saudi Arabia). The results are summarized in the following tables.

**Table A5:** CFS: value at baseline and at each post-baseline visit—PPS (*n* = 84)—descriptive analysis by subgroup.

		Comfort Shield		Control	
		Desert (*n* = 6)	Europe (*n* = 38)	Desert (*n* = 7)	Europe (*n* = 33)
value at baseline	*n*	6	38	7	33
	mean (sd)	3.79 (0.71)	4.04 (0.52)	4.11 (0.61)	4.30 (0.55)
	median (iqr)	3.62 (3.37, 4.05)	3.94 (3.59, 4.39)	3.95 (3.60, 4.56)	4.41 (3.80, 4.65)
	min, max	3, 5	3, 5	3, 5	3, 5
	missing	0	0	0	0
value at week 4	*n*	5	36	6	32
	mean (sd)	3.37 (0.89)	3.89 (0.82)	2.22 (1.53)	3.72 (1.05)
	median (iqr)	3.74 (2.81, 4.09)	4.00 (3.43, 4.54)	2.15 (1.41, 3.36)	3.93 (3.25, 4.48)
	min, max	2, 4	1, 5	0, 4	0, 5
	missing	1	2	1	1
value at week 8	*n*	6	35	6	32
	mean (sd)	3.79 (1.30)	3.94 (0.97)	2.21 (1.91)	3.88 (0.83)
	median (iqr)	4.20 (2.60, 4.84)	4.31 (3.38, 4.68)	2.56 (0.58, 3.22)	3.99 (3.37, 4.45)
	min, max	2, 5	0, 5	0, 5	2, 5
	missing	0	3	1	1

**Table A6:** CFS: value at baseline and change from baseline to each post-baseline visit—PPS (*n* = 84)—descriptive analysis by subgroup.

		Comfort Shield		Control	
		Desert (*n* = 6)	Europe (*n* = 38)	Desert (*n* = 7)	Europe (*n* = 33)
baseline	*n*	6	38	7	33
	mean (sd)	3.79 (0.71)	4.04 (0.52)	4.11 (0.61)	4.30 (0.55)
	median (iqr)	3.62 (3.37, 4.05)	3.94 (3.59, 4.39)	3.95 (3.60, 4.56)	4.41 (3.80, 4.65)
	min, max	3, 5	3, 5	3, 5	3, 5
	missing	0	0	0	0
change from baseline to week 4	*n*	5	36	6	32
	mean (sd)	−0.43 (0.59)	−0.18 (0.78)	−1.74 (1.16)	−0.57 (0.90)
	median (iqr)	−0.70 (−0.91, −0.06)	−0.03 (−0.52, 0.31)	−1.51 (−2.29, −0.82)	−0.35 (−0.92, −0.14)
	min, max	−1, 0	−3, 1	−4, −1	−4, 1
	missing	1	2	1	1
change from baseline to week 8	*n*	6	35	6	32
	mean (sd)	0.00 (0.98)	−0.14 (1.06)	−1.79 (1.48)	−0.42 (0.82)
	median (iqr)	−0.02 (−0.62, 0.64)	−0.07 (−0.56, 0.56)	−1.33 (−3.11, −0.75)	−0.23 (−0.64, 0.12)
	min, max	−1, 1	−4, 1	−4, 0	−3, 1
	missing	0	3	1	1

**Table A7:** OSDI: value at baseline and at each post-baseline visit—PPS (*n* = 84)—descriptive analysis by subgroup.

		Comfort Shield		Control	
		Desert (*n* = 6)	Europe (*n* = 38)	Desert (*n* = 7)	Europe (*n* = 33)
baseline	*n*	6	38	7	33
	mean (sd)	62.34 (18.55)	56.63 (16.29)	62.98 (20.34)	60.73 (16.53)
	median (iqr)	63.63 (51.14, 76.14)	54.36 (43.32, 68.44)	70.45 (46.93, 77.71)	61.11 (52.08, 75.00)
	min, max	35, 84	34, 92	36, 85	34, 95
	missing	0	0	0	0
value at week 4	*n*	6	38	7	33
	mean (sd)	36.89 (31.71)	43.75 (18.39)	49.74 (15.73)	56.71 (20.37)
	median (iqr)	20.45 (19.18, 61.36)	40.91 (32.71, 55.31)	43.18 (36.82, 64.02)	55.56 (41.67, 69.44)
	min, max	7, 80	9, 85	33, 70	22, 95
	missing	0	0	0	0
value at week 8	*n*	6	38	7	33
	mean (sd)	26.45 (20.34)	42.95 (18.26)	65.85 (12.65)	54.11 (22.42)
	median (iqr)	32.95 (9.38, 40.91)	41.67 (29.27, 55.94)	65.91 (58.62, 74.75)	52.08 (35.42, 75.00)
	min, max	0, 48	15, 88	46, 82	9, 93
	missing	0	0	0	0

**Table A8:** OSDI: Value at baseline and change from baseline to each post-baseline visit—PPS (*n* = 84)—descriptive analysis by subgroup.

		Comfort Shield		Control	
		Desert (*n* = 6)	Europe (*n* = 38)	Desert (*n* = 7)	Europe (*n* = 33)
baseline	*n*	6	38	7	33
	mean (sd)	62.34 (18.55)	56.63 (16.29)	62.98 (20.34)	60.73 (16.53)
	median (iqr)	63.63 (51.14, 76.14)	54.36 (43.32, 68.44)	70.45 (46.93, 77.71)	61.11 (52.08, 75.00)
	min, max	35, 84	34, 92	36, 85	34, 95
	missing	0	0	0	0
change from baseline to week 4	*n*	6	38	7	33
	mean (sd)	−25.45 (33.48)	−12.88 (18.03)	−13.24 (12.94)	−4.02 (12.19)
	median (iqr)	−34.59 (−45.60, −7.07)	−9.66 (−19.69, −2.61)	−12.50 (−16.32, −5.93)	−2.50 (−8.61, 0.00)
	min, max	−64, 27	−71, 17	−38, 2	−31, 26
	missing	0	0	0	0
change from baseline to week 8	*n*	6	38	7	33
	mean (sd)	−35.89 (27.94)	−13.68 (20.02)	2.88 (18.26)	−6.63 (16.47)
	median (iqr)	−34.95 (−40.34, −23.15)	−12.50 (−20.98, −0.23)	−2.50 (−7.41, 4.52)	−3.86 (−18.75, 6.82)
	min, max	−84, 0	−69, 27	−12, 41	−43, 15
	missing	0	0	0	0

## Appendix H. Fluctuation of Corneal Fluorescein Staining in the Control Group

The HYLAN M study included patients with severe dry eye, whose optimum therapy had not changed within the two months (in the case of concomitant cyclosporine therapy, three months) prior to inclusion. Therefore, it was assumed that the corneal fluorescein staining in the control group, where the therapy remained unchanged, will remain fairly constant over the eight-week study period. To prove this assumption, we post hoc analyzed the changes in corneal fluorescein staining of the study eyes of the control group from the baseline to the week 4 visit and from the week 4 to the week 8 visit. We found that from the baseline visit to the week 4 visit, the CFS Oxford grade improved on average by 0.69 with a standard deviation of 1.05, and from the week 4 visit to the week 8 visit, it worsened on the average by 0.23 with a standard deviation of 1.12. An improvement from the baseline to the week 4 visit might be attributable to the fact that the patients participated in a study with controlled dropping frequency and, therefore, adhered more strictly to the prescribed therapy. However, this argument should not apply to the differences observed between the week 8 visit and the week 4 visit.

We interpret our findings in the sense that CFS as an endpoint is subject to significant fluctuation in patients suffering from severe dry eye disease.
